# The mechanisms and processes of connection: developing a causal chain model capturing impacts of receiving recorded mental health recovery narratives

**DOI:** 10.1186/s12888-019-2405-z

**Published:** 2019-12-21

**Authors:** Fiona Ng, Ashleigh Charles, Kristian Pollock, Stefan Rennick-Egglestone, Pim Cuijpers, Steve Gillard, Lian van der Krieke, Rob Bongaardt, Scott Pomberth, Julie Repper, James Roe, Joy Llewellyn-Beardsley, Caroline Yeo, Ada Hui, Laurie Hare-Duke, David Manley, Mike Slade

**Affiliations:** 1 School of Health Sciences, Institute of Mental Health, University of Nottingham, Nottingham, UK; 2 School of Health Sciences, University of Nottingham, Nottingham, UK; 3 Department of Clinical, Neuro & Developmental Psychology, Amsterdam Public Health Research Institute, Vrije Universiteit, Amsterdam, Netherlands; 4 Population Health Research Institute, St. George’s University of London, London, UK; 5 University Medical Center Groningen, University Center of Psychiatry, University of Groningen, Groningen, Netherlands; 6 Department of Health, Social and Welfare Studies, Faculty of Health and Social Sciences, University of South-Eastern Norway, Porsgrunn, Norway; 7 Nottinghamshire Healthcare NHS Foundation Trust, Nottingham, UK; 8 Implementing Recovery for Organisational Change (ImROC), Nottingham, UK; 9 National Institute for Health Research, ARC East Midlands, University of Nottingham, Nottingham, UK

**Keywords:** Causal chain model, Mental health, Recovery, Narrative, Recovery narrative, Recovery story, Connection, Qualitative

## Abstract

**Background:**

Mental health recovery narratives are a core component of recovery-oriented interventions such as peer support and anti-stigma campaigns. A substantial number of recorded recovery narratives are now publicly available online in different modalities and in published books. Whilst the benefits of telling one’s story have been investigated, much less is known about how recorded narratives of differing modalities impact on recipients. A previous qualitative study identified connection to the narrator and/or to events in the narrative to be a core mechanism of change. The factors that influence how individuals connect with a recorded narrative are unknown. The aim of the current study was to characterise the immediate effects of receiving recovery narratives presented in a range of modalities (text, video and audio), by establishing the mechanisms of connection and the processes by which connection leads to outcomes.

**Method:**

A study involving 40 mental health service users in England was conducted. Participants were presented with up to 10 randomly-selected recovery narratives and were interviewed on the immediate impact of each narrative. Thematic analysis was used to identify the mechanisms of connection and how connection leads to outcome.

**Results:**

Receiving a recovery narrative led participants to reflect upon their own experiences or those of others, which then led to connection through three mechanisms: comparing oneself with the narrative and narrator; learning about other’s experiences; and experiencing empathy. These mechanisms led to outcomes through three processes: the identification of change (through attending to narrative structure); the interpretation of change (through attending to narrative content); and the internalisation of interpretations.

**Conclusions:**

This is the first study to identify mechanisms and processes of connection with recorded recovery narratives. The empirically-based causal chain model developed in this study describes the immediate effects on recipients. This model can inform selection of narratives for use in interventions, and be used to support peer support workers in recounting their own recovery narratives in ways which are maximally beneficial to others.

## Background

Contemporary understandings of recovery extend the clinical focus on symptomatic remission to incorporate personal understanding about the processes of recovery, in which individuals are experts of their own lived experiences [1, 2]. Consistent with national [3–5]and international [6] mental health policy, recovery is defined as a personal process of living with or without the mental health concerns [7], which includes elements of connectedness, hope, identity, meaning and empowerment [8]. Mental health systems, internationally, have increasingly adopted a recovery-oriented approach to servicing [9–11].

Recovery-oriented approaches have seen the introduction of new interventions, which endorse the central importance of experiential knowledge, such as the sharing of personal recovery narratives. These are defined as a first person lived-experience accounts of mental health concerns which include both aspects of struggle/adversity and survival/strength [12, 13]. Personal recovery narratives are increasingly used in clinical interventions, public health campaigns, and as part of the peer support worker role in mental health systems. Illustrative examples of three well-established interventions which use narratives are now described.

Narrative Enhancement Cognitive Therapy (NECT) is a clinical intervention which addresses self-stigma for people with mental health problems [14]. Self-stigma involves strong group identification and the internalisation of negative stereotypes by an individual [15] and has been identified to have negative effects on an individual’s self-esteem, self-efficacy, and treatment participation. NECT aims to address self-stigma through three mechanisms: psychoeducation to redress negative stereotypes; cognitive restructuring through the provision of adaptive coping strategies; and narrative enhancement through the facilitation of insight to assist individuals to make meaning of their experiences [14, 16]. Therefore, changes to an individual’s self-narrative are theorised to reduce self-stigma. Randomised controlled trial evaluations of NECT have shown a positive impact on self-stigma, and associated outcomes such as hope, self-esteem and quality of life [17, 18].

Public health anti-stigma campaigns, such as Time to Change [19] in England, work under the premise that having social contact with individuals who have lived experience of mental health concerns who share these experiences will reduce public stigma through improving knowledge, attitudes and behaviour [20]. Disclosure of one’s mental health status is a core component of social contact, which can occur in face to face settings or through accessing online recorded narratives [21, 22]. In a study accessing the changes in public stigma in England, survey data was collected from approximately 1700 individuals each year, spanning ten-years (2003–2013). Findings indicated that there were increases in positive attitudes in terms of prejudice and a reduction in exclusion towards people with mental health concerns [19]. Disclosure of mental health concerns through social contact was found to reduce public stigma, and has been associated with lower levels of self-stigma and higher rates of help-seeking behaviours and treatment utilisation [23].

Despite the increasing evidence for the effectiveness of interventions which utilise narratives of individuals with lived experience of mental health concerns, the evidence base for the mechanisms by which narratives impact on recipients is limited. The best evidence base comes from our third example, which concerns peer support workers. Peer support worker roles use the experiential knowledge of individuals who are in recovery from mental health concerns to provide support to others [24]. The use of experiential knowledge includes when a peer support worker shares their own experienced difficulties to the person they are supporting. A change model of peer support interventions identified three core mechanisms: building trusting relationships based on lived experience; role-modelling recovery; and assist in engaging with clinicians, services, and the community [25]. The effectiveness of peer support interventions has been identified in a Cochrane review to be equivalent to services provided by mental health professionals [26].

Evidence about the impact of recorded recovery narratives has only recently emerged. We have undertaken a series of studies to understand the impact of recorded recovery narratives on recipients, of which the current paper is the third study. First, we conducted a systematic review and narrative synthesis to develop a conceptual framework to characterise the impact of live or recorded recovery narratives on recipients [27]. Five publications were included, and the synthesis identified six broad potential impacts of narratives: connectedness; understanding recovery; reduction in stigma; validation of personal experiences; emotional response; and behavioural responses. Each impact was identified to be helpful or unhelpful, and could be moderated by the characteristics of the recipient, context and narrative. However, there were large methodological differences between the included studies. All studies were diagnosis specific, with an emphasis on understanding the effects of engaging in narratives featuring eating disorder behaviours. A sub-group analysis identified harmful disorder specific impacts through the emulation of eating disorder behaviour which contributed to the maintenance of the disorder. Additionally, the range of modalities of recovery narratives used within studies included in the systematic review was narrow. These included; a narrative read out by the researcher, a written memoir, video narratives, and spoken stories as part of a telling my story course [21]. Each of these modalities were evaluated separately and the effect of receiving multiple narrative modalities is unknown. The synthesis was therefore limited in its generalisability.

Secondly, we conducted a qualitative interview study involving 77 participants with mental health concerns [28]. The aim was to develop a preliminary trans-diagnostic change model characterising the range of possible impacts of recovery narratives and how they occur. Participants were recruited from four under-represented groups within mental health services: those with experience of psychosis who have not used mental health services for the last 5 years; black and minority ethnic groups; those who have experienced difficulties accessing mental health services; and peer workers. Iterative thematic analysis was used to develop the preliminary change model. Impact primarily occurs when the narrative recipient develops a connection to a narrator and/or their narrative, and is mediated by the recipient recognising shared experiences, noticing narrator achievements, noticing narrator difficulties, learning how recovery happens, or experiencing an emotional release. Helpful outcomes of receiving recovery narratives comprised hope, connectedness, validation, empowerment, appreciation, reference shift and stigma reduction. Impact was moderated by the perceived authenticity of the narrative, and whether the recipient was experiencing a crisis.

The study had several limitations. The preliminary framework captured impact over a longer duration, as participants recalled narratives which they encountered over the past few years or decades. Whilst this provides an indication of the long-term impact, it cannot provide understanding of how individuals may be immediately impacted by recovery narratives. Understanding of impact over a longer duration may introduce the risk of recall bias, where the recall of specific details of narrative impact may be influenced by other experiences. Connection was identified as the core mechanism of change in the study, but specific mechanisms and processes underpinning connection were not characterised. Finally, the narratives recalled by participants comprised both live and recorded recovery narratives, so the results may not be specific to the impact of recorded recovery narratives.

A knowledge gap exists in relation to understanding the immediate impact of recorded recovery narratives across different modalities. The aim of this study is to refine preliminary change models presented through previous studies [27, 28] in order to produce a testable causal chain model characterising how receiving a recorded mental health recovery narrative impacts on connection. The objectives are to understand the mechanisms by which connection with narrative and narrator characteristics occurs (Objective 1) and to characterise the processes by which these mechanisms of connection lead to outcomes (Objective 2).

## Method

This interview study was conducted as part of the Narrative Experiences Online (NEON) Programme (researchintorecovery.com/neon), which is investigating whether engagement with recorded mental health recovery narratives can influence an individual’s recovery journey. Ethical Committee approval was obtained (London-West London REC and GTAC 18/LO/0991) and all participants gave written informed consent. Findings will inform a future clinical trial (ISRCTN11152837).

### Participants

Eligible participants were people with current mental health concerns, using statutory mental health services, aged over 18 years-old, able to provide informed consent, and fluent in English. Individuals who were experiencing crisis or who were otherwise unable to take part in the research were excluded. Participants were recruited from statutory mental health services within one Healthcare Trust in the East Midlands of England.

The study was promoted as an investigation of narrative impact, through social media, advertisements within services (e.g. posters and newsletters), and by clinicians and managers from Improving Access to Psychological Therapy Services, community forensic services, locality mental health teams, and recovery colleges. Both clinician referrals and self-referrals to the study were accepted. Potential participants received the study’s participant information sheet from their clinician or directly from the researchers. Interested participants then contacted researchers or gave their clinician permission to pass on their contact details. Researchers assessed eligibility and informed consent was obtained prior to the interview. Interviews took place on a university or clinical health service premise.

### Procedures

The NEON Collection is a managed set of recorded mental health recovery narratives for which organisations or individuals have provided permission for use [29]. A total of approximately 680 narratives was part of the NEON collection at the time of this study, drawn from four external collections and represented narratives from six countries. Recovery narratives were defined as a first person account of lived experience that includes elements of both adversity/struggle and of strength/success/survival, and refers to events or actions over a period of time. The inclusion criteria for recovery narratives in the wider NEON Collection were: 1) fitting the definition of a recovery narrative, 2) available in a digital media file (audio, text, video, HTML, image), 3) informed consent was provided for use, and 4) the narrative is presented in English. Exclusion criteria were; fictional, describing criminal activity, containing defamatory material, media of low quality, and narrative that contain detailed description of harmful behaviour (e.g. specific techniques associated with self-harm, eating disorders, suicide).

A sub-set of 30 narratives were assembled from the NEON Collection by two researchers. Narratives were purposively selected to maximise variation in three dimensions: modality, narrator diversity and length [30]. Multimedia use in educational settings has been shown to increase depth of learning in students [31], suggesting that this may promote engagement and cater to different learning styles within individuals. Narratives with a substantial range of modality are available in the public domain and the use of multimedia may also promote inclusiveness of individuals who experience disabilities or may have difficulty comprehending a specific mode of media, for example due to dyslexia. To accommodate, a mixture of text, audio and video-based narrative modalities were chosen. The selected narratives were diverse in narrator age, gender and ethnicity, given preliminary evidence that sociodemographic characteristics can influence connection [27]. Finally, to vary cognitive demands on participants, the chosen narratives were different in length. Text narratives ranged from half a page to three pages, video narratives ranged from one to 5 min, and audio narratives ranged from two to 3 min (see Additional file 1 for characteristics of all included narratives). Based on a pilot of the study protocol, it was estimated that on average participants would take no longer than 10 min to read, watch or listen to a narrative. Overall, 15 text (comprising poems and prose text), 10 video and five audio-based narratives were included in the study. Narratives included in the study were introduced to participants as mental health recovery narratives which contained aspects of survival and struggle, occurring over a period of time.

All participants were asked about their stage of recovery in alignment with stages identified by Llewellyn-Beardsley and colleagues [12] and completed the Herth Hope Index (HHI). The HHI is a 12-item abbreviated measure adapted from the Herth Hope Scale [32]. Developed for use in clinical settings, each item is self-rated on a four-point scale from ‘strongly disagree’ (low hope) to ‘strongly agree’, and the total score ranges from 12 (low hope) to 48. The HHI has good psychometric properties with high internal consistency (Cronbach alpha = 0.97), reliability (0.91) and content validity [32]. Participants were asked whether they had any disabilities, which would preclude them from engaging with specific types of narratives, for example arising from visual, hearing or learning disabilities. If individuals expressed preferences, then a random selection of ten stories drawn from the stories consistent with those preferences was provided if possible. If no preferences were given, then a random selection of ten stories from all 30 stories was provided. As far as consistent with preferences, a mix of text, audio and video-based narratives were selected. Following the completion of the demographic questionnaire, participants were iteratively shown up to ten stories, but fewer if requested. After engaging with each narrative, participants provided qualitative feedback on three questions: How connected to the story did you feel? How connected to the narrator did you feel? and How hopeful did the story make you feel? Participants were prompted discuss reasons for why they felt connected or hopeful as a result of engaging in the narrative. Further prompts from the researcher were guided by participant responses. Where participants experienced distress as a result of engaging with a recovery narrative, the researcher paused the interview to support the participant and ask whether they wanted to continue with the interview. Participants were reimbursed £20 and travel expenses. All interviews were audio recorded and transcribed verbatim.

### Analysis

The analysis took an interpretative approach. To address objective 1 (mechanisms of connection) an inductive and deductive approach to qualitative analysis was used [33], to build on the preliminary evidence on the mechanisms of connection derived from previous studies. To address objective 2 (impact of connection on outcomes) an inductive approach was used to investigate the processes by which connection mechanisms lead to outcome, due to the absence of any empirical evidence on this component of the causal chain.

A preliminary coding framework, used as part of the deductive approach in objective 1, was developed through the synthesis of existing research on the impact of receiving recovery narratives [27, 28]. This is shown as Additional file 2. The preliminary coding framework was then refined through a thematic analysis of the interview data, guided by a six-step process outlined by Braun and Clarke [34]. First, data from the semi-structured interviews were transcribed verbatim and anonymised. Secondly, two analysts (FN, AC) familiarised themselves with the data by reading the transcripts and preliminary coding frameworks. Third, the two analysts independently coded the first four transcripts to refine the coding framework, and additional codes that did not fit the preliminary coding framework were generated to capture new themes. Fourth, discussions between analysts were held to develop the working coding framework. Discussions focused on the refinement of codes and definitions, where all four transcripts were discussed in full. Fifth, the coding framework was discussed with the wider analyst team consisting of nine analysts, to further refine the framework and identify disagreements, which were resolved via consensus. Sixth, the revised coding framework was applied to the remaining transcripts by the two initial analysts. The causal chain model was developed from the synthesised themes identified in the coding framework. Specific attention was made to understand how connection occurs (mechanisms) and how connection leads to outcomes (processes). To evaluate the fit of the causal chain model, two participant transcripts were reviewed in light of the developed model by one researcher. The wider analyst team had expertise in psychology, nursing, health services research, computer science, mad studies, and qualitative research. Several analysts also had lived experience of mental health concerns, enhancing the role of lived experience in the analysis and interpretation of findings [35]. A sub-group analysis was conducted on data collected from narratives, which caused participants to feel distressed. The purpose of the sub-group analysis was to identify the narrative characteristics, which contribute to distress, in order to increase awareness of the characteristics which may lead to unhelpful outcomes. A thematic analysis was conducted through extracting participant responses to the specific narrative which elicited distress. Participants were considered distressed if they requested a pause in the interview due to an emotional or physical response to the narrative. All analysis was conducted using NVivo version 12.

## Results

### Participant characteristics

The characteristics of the 40 participants are shown in Table 1.

**Table 1:** Participant characteristics (*N* = 40)

Characteristic	n (%)
Age (years) mean (SD, range)	44.5 (16.7, 18 to 76)
Gender	
Female	24 (60)
Male	16 (40)
Ethnicity	
White	33 (82.5)
Black/African/Caribbean/Black British	1 (2.5)
Asian/Asian British	5 (12.5)
Other	1 (2.5)
Sexual Orientation	
Heterosexual	29 (72.5)
LGBT+	10 (25)
Prefer not to say	1 (2.5)
Educational Attainment	
No qualification	3 (7.5)
GCSEs or equivalent	6 (15)
A-Levels/AS-levels/NVQ or equivalent	12 (30)
Degree level qualification	14 (35)
Higher degree level qualification	4 (10)
Other	1 (2.5)
Relationship Status	
Single	26 (65)
In a relationship	14 (35)
Diagnosis	
Schizophrenia or other psychosis	9 (22.5)
Bipolar disorder	7 (17.5)
Mood disorder	14 (35)
Personality disorder	8 (20)
Other	2 (5)
Recovery Stage	
I am recovered	1 (2.5%)
I am living well	4 (10%)
I am making progress	18 (45%)
I am surviving day to day	17 (42.5%)
Herth Hope Index mean (SD, range)	31.1 (5.3, 17–40)
Participant has engaged in a recovery narrative in the past year (yes)	23 (57.5%)
Participant has told their own recovery narrative to others in the past (yes)	24 (60%)
Participants indicating a preference for narratives (yes)	12 (30%)

Scores on the Herth Hope Index indicate that participants had a moderate level of hope at the start of the interview, with the majority of participants indicating that they were at an earlier stage of recovery (I am making progress or surviving day to day). Of the individuals who indicated a preference for narratives, 10 individuals indicated one preference, whilst two individuals indicated two preferences. Five participants indicated a preference for not reading text or listening to audio-based narratives respectively. The remaining four participants indicated a preference for not watching video narratives. Explanations for preferences included difficulties with concentration, the ability to gain non-verbal cues, and individual preferences for a specific modality. Participants on average engaged with 7.1 narratives (SD = 2.0, Median = 7).

### Causal chain model

**Fig. 1:**
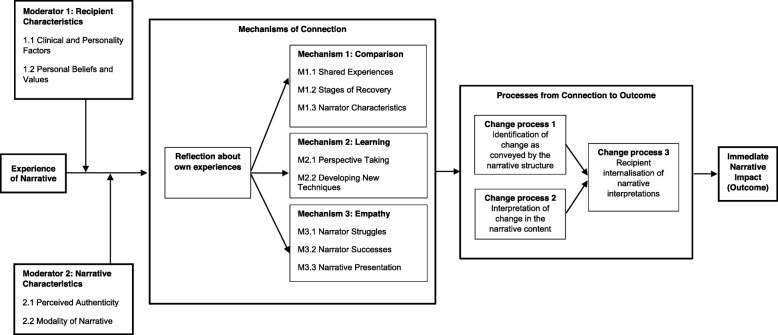
Causal chain model for the immediate effects of receiving a recorded mental health recovery narrative

Findings from the interviews were thematically analysed and the emergent themes was used to construct the causal chain model. The aid readability we first present the summarised causal chain model (Fig. 1), then descriptions of each theme. The causal chain model demonstrates that the impact of a narrative is moderated by both recipient characteristics (e.g. clinical, personality) and narrative characteristics (modality and perception of authenticity). Three mechanisms of connection; comparison, learning, empathy – all begin with the participant’s reflection about their own experiences. These mechanisms led to impact through identification of change in the narrator as conveyed by the narrative structure or interpretation of change in the narrative content, both of which lead to internalisation of the interpretation of the narrative.

### Moderating factors

Two moderating factors, recipient and narrative characteristics were identified.

#### Moderator 1: recipient characteristics

The effect of different narratives on participants varied due to individual differences. Socio-demographic, clinical, personality factors, and one’s beliefs and values were identified by participants to influence the manner in which they engaged with a recovery narrative.

##### Clinical and personality factors

Self-reported clinical factors discussed by participants included their ability to concentrate, as side effects from medication and the experience of mental health symptoms (such as hearing voices) affected their engagement. Self-reported personality factors included having general difficulties with connecting with others.

*‘As a person, I don’t care about people. I am not a people person, very difficult to get involved personally with anybody’ (#15)*

##### Personal beliefs and values

Recipient beliefs and values refer to the world-view a participant holds and how this is influenced by their prior experiences and perspectives. Recipients’ beliefs and values were reported to influence the interpretations of experiences depicted in the narrative. Narratives which were perceived to be ambiguous, were interpreted through a lens which fitted participant’s pre-existing models of understanding.

*‘When it says ‘who recovers and doesn’t is inequality at large’ … that speaks to me quite a lot. I had quite negative experience with mental health teams when I first came to this and ended up paying privately for my first diagnosis’ (#4)*

#### Moderator 2: narrative characteristics

##### Perceived authenticity

Narratives perceived as factual accounts contributed to its authenticity, resulting in a sense of connection, genuineness, and greater understanding of the experiences of the narrator. The inclusion of specific details of experience and honesty portrayed by narrators allowed for a more nuanced view. This was further exemplified by descriptions of the narrator’s emotional state as it allowed for parallels to be drawn between the narrator and the recipient’s experience.

‘*What she’s talking about is something that’s really hard to admit and the fact that she was sharing it with other people, that’s what makes you connect with somebody’ (#4)*

Misalignment between recipient and narrator views reduced a narrative’s perceived authenticity. Video and audio narratives were described as less genuine when limited emotional expression by the narrator. This was further exemplified in narratives considered superficial, for example, those using metaphors in which participants deemed to be cliché.

*‘It doesn’t feel as authentic … just the whole flower thing I think most of all because it’s such a cliché.’ (#14)*

Some participants also tried to deduce the motivation of the narrator to share their story. Narratives which portrayed the narrator’s desire to assist others were reported to be more authentic.

##### Modality of narrative

Most participants did not have difficulties with the narrative format. Some participants reported difficulties with concentration when engaging with text or audio-based narratives as there were no visual elements to focus on, and found video-based narratives to be more engaging. Visual and audio elements provided a more personal presentation, where participants had access to non-verbal cues and could make interpretations based on a narrator’s expression or mannerisms. These interpretations had a mixed effect on connection, and were related to perceived similarities between the narrator and the participant. In text-based narratives, visualisation of the narrative or narrator facilitated comparison. However, this was influenced by the clarity of expression and level of description within the narrative, and the interpretations made by the participants.

*‘The mind is a very powerful thing and I’m reading the story and I can imagine what has happened and how devastated she’s been and the issues that she’s been through and in lots of ways the lack of support that she’s felt’ (#10)*

The narrative form referred to the specific format in which the narrative was presented, with poems and prose most commonly discussed. Prose-based texts were considered more accessible for comprehension, whilst poems had a mixed effect on participants. Whilst some participants reported difficulties with comprehension, others reported the benefits of metaphors provided in conveying experience. Providing varying lengths of narratives was considered helpful, however some participants preferred shorter ones. Whilst shorter narratives made fewer demands on concentration, they also risked a lack of content necessary to compare oneself with others to or to learn from their experience.

*‘Because its poetry there’s a lot you don’t know about the narrator and it’s not the same as a prose narrative. You just sort of get an impression of how he’s feeling.’ (#2)*

### Objective 1: mechanisms of connection

#### Reflection of own experiences

The reflection of one’s experiences or the recollection of memories that paralleled experiences discussed in narratives was a core component of responding to a narrative. A participant’s self-reflection about their experiences influenced the mechanisms of connection and processes from connection to outcomes. Participant engagement and outcomes as a result of receiving narratives were related to the experiences a participant brought to the task.

*‘It could have been me writing it … there was one which mentioned suicide … that just brought my memories up. Wow that’s not a good thing, but at the time you’re in so much distress and emotional turmoil and pain.’ (#1)*

#### Mechanism 1: comparison

Comparison occurred between participants’ subjective evaluations of the characteristics of a narrative or narrator, which led to both connection with and disconnection from the narrative or narrator. Table 2 describes the specific identified types of comparison, and how these impact on connection.

**Table 2:** Types of comparison and their impact on connection

Types of comparison	Impact on connection + = positive impact - = negative impact
Shared Experiences	
Recognising shared experiences	
Coping strategies	+/−
Emotional experiences	+
Meaningful participation	+/−
Mental health support	+/−
Past experience or previous self	+/−
Progress in recovery	+/−
Relationships	+/−
Stage of Recovery	
Comparison in stage of recovery	
Upward comparison	+/−
Downward comparison	+
Narrator Characteristics	
Similarities in characteristics	
Age	+/−
Gender	+/−
Life experiences	+/−
Diagnosis	+/−
Thought processes	+/−
Country	+/−
Personal background	+/−
Culture and Religion	+/−

##### Shared experiences

The identification of shared experiences promoted a sense of relatedness. Shared experiences commonly identified are listed in Table 2 and were not limited to a participant’s own experiences. Reminders of experiences of others (for example family members or friends) also facilitated comparison. However, identifying shared experiences had the ability to leave participants feeling vulnerable, particularly when participants were currently having difficulties. For some, differences in experiences resulted in disconnection due to difficulties relating to the narrative or narrator. Other participants talked about gaining understanding or increasing self-awareness from understanding the differences.

*‘There is a sense of anxiety in that I want to remain abstinent, and reading stories about people drinking is possibly going to make me vulnerable’ (#38)*

##### Stage of recovery

Comparisons based on their stage of recovery were also reported in two forms. First, downward comparison when the participant judged they are further along in their recovery journey or had a better experience than the narrator. Second, upward comparison where the participant judged that the narrator is further along in their recovery or had a better experience than the participant.

Downward comparison led to a sense of empathy, as some participants could connect with the experiences and emotions of the narrator. Some participants explained that they received a sense of validation and perspective when they perceived that their experiences could have been much worse in light of receiving the narrative of others. However, the recency of an experience also affected the interpretations of comparisons made. Experiences which recently occurred facilitated connection, whilst experiences deemed to be no longer relevant hindered connection. Upward comparisons had the potential to induce positive or negative effects in participants. Positive effects included the sense of hope generated as a result of the narrator acting as role model for the possibility of recovery. Some participants, experienced a negative effect when they perceived the narrator’s achievements were unrealistic for them to achieve.

*‘The only hope it gave me was that I have got an advantage over that lady because the people trying to help me are speaking the same language, so I haven’t got that language problem … it is difficult to make yourself understood when you have got things you can’t deal with … to explain that to someone else it is really hard, even in your own language.’ (#30)*

##### Narrator characteristics

Comparisons in personal characteristics provided participants with an indication of how similar their experiences were to those of the narrator. Common comparisons made included; age, gender, life experiences, diagnosis, thought processes, country, personal background, and culture/religion. Whilst disparity in personal characteristics did not necessarily lead to disconnection, some participants reported difficulty in relating to such narratives. For example, participants reported difficulties relating to narratives where the narrators had a different accent or indicated experiences of a different health system.

*‘I’d say the story is mirroring me and it’s helpful to know that somebody else, especially because I’ve been a Christian for so many years and people find that helps them in their recovery … ’ (#7)*

#### Mechanism 2: learning

Learning from the narrative or narrator was reported to facilitate learning in two ways; first through perspective taking and second the development of new techniques. The narrative content facilitated learning through the presentation of perspectives or techniques which had not previously been encountered or were considered interesting to the participant. Table 3 outlines the factors that influence learning and whether these had a positive or negative impact on connection.

**Table 3:** Influences that affect learning and their impact on connection

Influences on learning	Impact on connection + = positive impact - = negative impact
2.1 Perspective Taking	
Understanding the impact of mental health concerns on others	+/−
Understanding own experiences	+
Learning about differing beliefs and values	+
2.2 Developing new techniques	
Managing treatment and services	
Discussions with mental health clinicians	+
Managing medication	+
Supporting daily living	
Coping strategies	+

##### Perspective taking

The description of the impact of mental health concerns that were different to the participant’s own experiences provided deeper understanding of how mental health concerns may affect others. Additionally, participants who considered themselves further ahead in their recovery journey, gained insight into difficulties which they may not have experienced themselves. For participants with difficulty articulating their own experiences, engaging in recovery narratives which reflected their experiences acted as a form of expression. Additionally, narratives exposed participants to differing beliefs and values which may contrast from their own.

*‘I’m learning about other people’s experiences a) because that provides comfort and b) because it kind of contextualises my own experiences, but also informed me on something specific.’ (#14)*

##### Developing new techniques

Narratives provided insights and widened understanding of the helpful techniques narrators engaged in to manage treatment/services and daily living. The presentation of what worked for narrators was reported to be helpful by participants as these provided ideas for what they could try to integrate into their own lives. Ways to converse with mental health clinicians, medication management, and the use of differing coping strategies were indicated as helpful by participants.

*‘It’s a good thing that she has also given her techniques of how to cope with negative thoughts, so that’s helpful, because I think when you relate to a story and they say things that have helped them, then that’s really good.’ (#9)*

#### Mechanism 3: empathy

Ninety percent of participants reported a sense of empathy towards at least one narrative or narrator. Empathy occurred because of the interaction between self-reflection, comparison, and learning. However participants also reported a sense of empathy from being interested or engaged in the narrative despite not having similarities or learning from the story. Empathy was described as the sharing of emotions arising from the narrative and included the emotional reactions of the recipient. Influences of empathy and their impact on connection are presented in Table 4.

**Table 4:** Influences that affect empathy and their impact on connection

Sources of Empathy	Impact on Empathy + = positive impact - = negative impact
3.1 Narrator Struggles	
Discussion of narrator struggles or adversity	
Diagnosis	+
Life circumstances	+
Difficulties with interpersonal relationships	+
Family experiences	+
Emotional experiences	+
3.2 Narrator Successes	
Recognition of recovery progress in the narrative	
Gaining control of life/self-determination	+
Positive engagement with mental health services	+
Participation in meaningful activities	+
3.3 Narrative Presentation	
Language	+
Tone	+/−
Mannerisms and expressions of the narrator	+/−

##### Narrator struggles

Narratives which described the struggles and adversity experienced by the narrator led to a stronger perception of empathy. Gaining insight into the difficulties of others was considered a privilege and provided perspectives which may not have been accessible before. A recognition of differences in experience did not preclude the generation of empathy. However, the portrayal of emotional experiences within narratives was a vital factor in supporting a participant’s sense of empathy. Narratives deemed to be lacking in description of the emotional experience of narrators were reported to be more difficult to connect with. Narratives which portrayed narrators as having made progress in recovery also facilitated a sense of empathy through the sharing of positive experiences.

*‘It’s a sad story. It’s totally not in my experience but I can understand it’s a sad story that hasn’t had a happy ending. It didn’t have the happy ending that she wanted, which was family relationships’ (#6)*

##### Narrator successes

Empathy elicited through the narrator’s successes was in recognition of recovery progress in the narrative and led to participants vicariously feeling positive for the narrator. The identification of success was often preceded by discussion of struggles the narrators had experienced. The identification of narrators gaining control of life or demonstrating self-determination was identified to elicit empathy and was particularly noted when narrators made active decisions to manage their mental health.

*‘I feel that the lady, at the beginning, I didn’t feel she thought she was in control of her life and the direction it’s going but by the end of it she decided to gain control back and let it go because it’s poison to yourself” (#1)*

##### Narrative presentation

The presence of descriptive language and the tone of the narrative supported the realistic portrayal of experiences and emotion of the narrator which facilitated empathy. For example, the use of humour was reported to promote empathy as it allowed for the portrayal of sensitive emotional experiences in a more approachable and light-hearted manner. However, some participants held the contrasting view, such that the use of humour was insensitive and demonstrated a lack of insight into their experiences. The mannerisms and expressions of the narrator, in video-based narratives, also provided additional contextual information. These could have a positive or negative effect on empathy.

*‘It made me a bit uncomfortable … he was more unsettled and it was a bit close to home, seeing him visually speak and noticing that he was he seemed a bit skittish, it instantly put me in that position.’ (#36)*

### Objective 2: processes from connection to outcome

The experience of connection had the potential to effect outcomes through three change processes, shown in Table 5. A recipient’s internalisation of change arising from the narrative occurred through either the identification of change in the narrative through the narrative structure, or the interpretation of change through the valence of the narrative content and presentation. Internalisation was defined as the application of change identified in recovery narratives to one’s own life.

**Table 5:** Processes by which connection mechanisms influences outcome

Processes from connection mechanism to outcome	Impact on outcome + = positive impact - = negative impact
Change process 1: Identifying the presence of change in a narrative	
Narrative Structure	
Upward trajectory	+
Disjointed	–
Incomplete/Not a recovery story	–
Turning point	+
Circular	–
Change process 2: Interpreting the valence of change depicted within a narrative	
Narrative Content	
Possibility of achievements	+
Lack of perceived progress	–
Empowerment of the Narrator	+
Narrative Modality	
Visual components	+/−
Change process 3: Recipient Internalisation of change depicted within a narrative	
Noticing own achievements	+
Pessimism about the possibility of recovery	–
Gaining validation from narrative or narrator	+
Optimism about human nature	+
Reframing of experience	+

#### Change process 1: identifying the presence of change in a narrative

Some participants identified change through the overall narrative structure, which provided insight into the progress made with recovery. Narratives which depicted an upward trajectory or a clear turning point, inferred that positive changes were possible, where the inclusion of a description of narrators’ success or achievements promoted a sense of hope in participants. Yet, pessimism was reported by participants when there was a lack of perceived change, for example in disjointed or circular narratives. Similarly, incomplete narratives which conveyed experiences at a single time point were noted to not provide sufficient insights to observe change. Participants questioned whether incomplete narratives could be conceptualised as a recovery narrative, despite the study only including narratives which referred to events and actions over a period of time.

*‘It was a snapshot of something bigger, I would prefer to hear the bigger, who is this person that’s talking, when were they treated, what happened next … ’ (#14)*

#### Change process 2: interpreting the valence of change depicted within a narrative

Interpreting the valence of change involved ascribing positive or negative interpretations was facilitated by the narrative content and the presentation. Narratives which depicted the possibility of recovery and achievement were interpreted to be beneficial. Whilst the definitions of achievement were unique to each participant, these were broadly defined as survival and success. The determination to live well, despite challenges associated with mental health concerns, or difficulties in accessing services was recognised as an expression of character-strength and personal agency. Narratives which portrayed the narrator as having limited progress in their recovery, were more likely to result in participants feeling pessimistic. Non-verbal cues provided by video-based narratives provided participants with additional contextual information on which to base their interpretations, which had positive and negative effects. Participants referred to the mannerisms and expressions of narrators as relevant to interpreting the valence of change, where a positive interpretation of change was identified in narrators who appeared hopeful (for example through smiling).

*‘I thought she [narrator] was pretty hopeful, seeing a big smile on her face when she said she’d performed her poetry. I mean that’s quite inspiring to see somebody have the determination to get control back in their life. She also appears to have hope for the future.’ (#1)*

#### Change process 3: recipient internalisation of change depicted in the narrative

The identification of and the interpretation of the valence of change led to recipients to apply these to their own life. The process of internalisation occurred in four different ways; noticing own achievements, gaining a sense of validation, becoming optimistic about human nature, becoming pessimistic about the possibility of recovery. Not all narratives were considered directly relevant by participants, and some noted not experiencing any internalisation of interpretations. Validation arose through the normalisation of a participant’s experiences and generated hope when narratives provided a sense that recovery was possible. Reflection also allowed participants to notice their own recovery achievements through recognition of their strength and progress. A minority of participants reported that narratives which described overcoming difficulties with the support of others, promoted optimism about human nature. However, pessimism about the possibility of recovery, was generated when negative interpretations of change aligned with a participant’s perception of their own recovery journey. Other participants reported that despite positive interpretation of change in the narrative, it did not necessarily lead to outcome. The narrative content was related to outcome, suggesting that there needs to be congruency between the narratives of the participant and narrator in order for a positive outcomes to occur.

*‘Being validated because sometimes you feel like you are the only one experiencing it so the fact that there are like 10 other stories out there where there is similar things being said, that really makes me feel more positive.’ (#9)*

### Sub-group analysis of narratives that caused distress

Five participants reported feeling distressed during the interview, following the experience of five different narratives. Two participants reported needing a break due to a pre-existing physical health condition. The remaining three participants reported feeling highly connected to the narrative which caused them distress and identified close parallels between their own and the narrator’s story. The impact of a narrative could be exacerbated through the use of emotionally descriptive language, and a strong sense of empathy experienced by the participant. This had a negative effect on connection, as reminders of past difficulties left participants feeling vulnerable and experiencing difficult emotions.

*‘Well I suppose just even the first line ‘haunted by the souls of the dead’ and … ah let’s not go there … and the whole story; been there, done that … it’s as far pessimistic as you can get’* (#6)

One participant also felt distress through witnessing the stigma experienced by the narrator, which resonated with their personal experiences. In this instance the impact of the narrative was seen to be exacerbated through the participant’s self-reflection of their own negative experiences which strengthened their sense of injustice over the manner in which the narrator was treated.

*‘ … it just makes me feel very sad … the thing was if somebody’s suffering from a mental health issue, they believe that the health professional are there to help and support them in a professional way. That doesn’t sound like that was there.’ (#10)*

## Discussion

This study examined the immediate effects of receiving mental health recovery narratives, to describe the mechanisms of connection and processes from connection to outcome. A testable causal chain model was developed through a thematic analysis of semi-structured interviews with 40 current mental health service users. Impact of narratives is mediated by recipient and narrative characteristics. Connection occurs firstly through the reflection of one’s own experiences, which leads to three mechanisms (comparison, learning, and empathy). These mechanisms of connection lead to outcomes through three processes 1) identifying the presence of change in the narrative, 2) interpreting the valence of change, and 3) the recipient internalising change. Short-term distress can arise when there is a strong sense of connection, and through the identification of parallels between the recipient, narrative or narrator.

### Relationship to prior research

The use of recovery narratives within mental health services has been argued to fulfil a neoliberal agenda [36]. The literature has called for the inclusion of diverse recovery narratives which represent differing trajectories and genres, including those that do not depict an upwards trajectory [12, 36]. In this study, the definition of a recovery narrative was deliberately broad to promote a diverse range of narratives [12]. However, narratives which were described as incomplete or only representing experiences at a single time point, led to questions over the conceptualisation of what is a recovery narrative. Whilst narratives perceived to convey an upward trajectory were reported to have a positive impact on outcomes. This may be suggestive of participant expectations for the type of recovery narrative they perceive to be helpful and may indicate differences between participants’ and the adopted definition in the current study. These expectations may have been influenced by participants’ prior engagement, such that more than half the sample had received a narrative in the past year and that the majority of participants indicated that they were at an earlier stage of recovery. The findings of this study only elicit the types of narratives that may be helpful and further research is required to understand whether narrative or recipient characteristics (such as age or gender) influences the helpfulness of a narrative. It should be highlighted however, these impacts do not imply that narrators should share their experiences using a prescribed format. Rather it indicates that a wide range of narratives is required in order to increase the possibility of connection occurring.

The effect of a narrative’s modality on outcomes have minimally been explored where prior research has predominately examined the impact of recovery narratives using one modality (for example the presentation of text-based narratives only) [37, 38]. Our findings indicate that the modality in which a narrative is presented may moderate the effects on connection. Visual and auditory cues provided within video and audio-based narratives were reported to provide greater context and assisted with a participant’s construction of a holistic view of the narrator. However, not all participants considered video or audio-based narratives as their preferred modality. Only a minority of participants indicated a preference for modality, yet this indicates that the provision of choice is an important aspect for interventions utilising recorded recovery narratives. The inclusion of a mix of modalities, including video, text, audio, image-based, and narrative forms, including narratives in poetry form, use imagery or metaphorical literary devices may increase the possibility that an individual will connect with at least one narrative.

Comparisons made between the narrative and participants’ stage of recovery, extends current understanding in the literature [27, 28]. At present the literature on recorded recovery narratives have indicated that stage of recovery, may moderate the impact of receiving a narrative. For example engaging with eating disorder narratives which provide specific examples of harmful behaviours might encourage individuals at an earlier stage of recovery to emulate these behaviours [39]. However, comparisons made in this study by participants focused on progress made in recovery, rather than behavioural expressions. In peer support worker interventions, role modelling was identified to promote feelings of optimism [25], yet the present study identified that upward comparison could lead to a mixed effect, such that a narrator’s achievements may be deemed to be too hopeful or unrealistic. Therefore, consideration of a potential gap in stage of recovery between what is portrayed within the narrative and where a recipient stands may be important when selecting recorded recovery narratives for use by individuals. Whilst it is difficult to predict reactions to specific narratives, understanding a recipient’s stage of recovery and life experiences may provide an indication of the relative acceptability of narratives.

Findings also indicate that comparisons made between the narrative and the participant did not necessarily have to be based on the participant’s own lived experiences, where comparison also occurs when narratives reminded recipients of the experience of others. These narratives left recipients with a sense of familiarity, and for some, generated a sense of empathy. This is an important finding and may indicate that recorded recovery narratives may have helpful effects on individuals who engage with people with mental health concerns, such as informal carers. Family members and other informal carers can play a significant role in the recovery of people with mental health concerns [40–42], and can also experience high levels of burden, distress, and stigma [43, 44]. The provision of recorded recovery narratives may be a low-cost approach to improving outcomes for family members and carers. Future research could test the applicability of the current model and the subsequent effects on outcomes in family members and carers of people with mental health concerns.

Recorded mental health recovery narratives are known to elicit emotional responses in recipients [28, 37]. However, minimal descriptions of empathy arising from receiving recorded recovery narratives are available. Recent studies have suggested that empathy is based on mirroring systems, whereby observing emotions from others may stimulate emotions in the observer [45]. Sharing one’s lived experience has been described to be a highly emotive experience [46], which may explain why, despite differences in experience, some participants experienced a sense of empathy towards the narrator. Although narratives included in this study portrayed both aspects of success and survival, witnessing negative experiences could cause distress in recipients. This may indicate that recipients focus their attention on aspects of narratives that resonate most strongly with them or reflect their current experiences. This may be an important consideration for interventions which feature recovery narratives, such that whilst narratives need to be relevant to a recipient, the tone of the narrative may need to match the recipient’s ability to process the narrative. Therefore, prior to the recommendation of specific narratives consideration of a participant’s current life experiences and potential triggers may be required.

Engaging in mental health recovery narratives has been found to improve a recipient’s understanding of recovery [28, 47] and providing participants with narratives depicting different experiences to their own could lead to learning. However, a negative impact on connection, due a lack of familiarity, can also occur. A balance between comparison and learning may need to occur when selecting narratives for recipients. It is unclear whether individuals value one mechanism of connection over another, or whether one mechanism is more important at differing stages of recovery, this might be an avenue for further research. The provision of randomly selected stories may facilitate the process of understanding what individual recipients may connect with. Yet, consideration over the readiness of recipients to receive material that may contrast to their personal beliefs or experiences might be valuable for clinicians who intend to use recorded recovery narratives in clinical practice.

The evaluative processes by which connection leads to impact have not previously been documented and further refines the causal change model. These have broader implications for interventions which feature narratives and for individuals who share their lived experiences with others. The refined causal change model can inform the selection of narratives for inclusion and provides insights into outcome variables which may be of interest in future intervention trials. Individuals who are preparing their lived experience to share with others could consider the narrative content and the structure to aid a recipient’s identification and interpretation of change. These recommendations are made with the intention to increase impact a narrative may have on others, rather than prescribing a specific manner for which narratives should comprise.

### Strengths and limitations

The strengths of this study include the study design, multiple use and range of narratives, and multiple analysts. First, the study design allowed for a more controlled approach to understanding impact, compared to receiving narratives within a live setting. Second, the use of multiple narratives (up to 10) spanning differing modalities, which may improve the acceptability and usability of the narratives by participants. This also allows for acknowledgement of the differing approaches narrators may wish to use to express their narrative and counters criticisms of the dialogic nature of narratives [12]. Third, given that the narrative content was identified to be important for the facilitation of connection, the inclusion of a range of narratives provides more opportunities for individuals to connect with a narrative.

The study, however, was not without limitations. First, the cultural background of participants may influence how narratives are interpreted. Participants in this study were predominately from a white background and have high levels of education, therefore responses may not fully encapsulate the perspectives of individuals from other population groups. Second, all narratives included in the study were relatively short; longer recorded recovery narratives, such as biographies, may have a different effect on recipients and could be a future research direction. Third, the study design did not allow for the follow up of participants, so it is unknown whether a participant’s level of connection was maintained, or whether participants had a delayed reaction to the narrative. Fourth, stage of recovery was also identified to influence the manner in which connection occurred. However, the present study used self-reported views of participants rather than objective measures of personal recovery. Future research could incorporate psychometrically validated measures to further understand the relationship between an individual’s recovery status and the impact of recovery narratives. Fifth, given the qualitative nature of the present study, more than one interpretation of the findings is possible. However, one strength of the study is the utilisation of multiple analysts with a range of expertise to reduce potential bias during the analysis process. Sixth, whilst the data reflected that the narrative modality had a moderating effect on connection, the data was inconclusive as to whether modality affects all participants in a specific manner. This may be due to the design of the study, where all individuals received a random set of narratives, as such cross-comparison of responses between narratives was not possible. Future work may involve giving the same set of narratives to a large number of diverse recipients, to identify individual narrative characteristics which have an overall positive impact.

## Conclusion

Recorded recovery narratives are increasingly available in the public domain, and this study provides an empirically-informed characterisation of the mechanisms and processes involved in generated outcomes in recipients. Connection to a narrative occurs through self-reflection, comparison, learning, and the experience of empathy in recipients, where recipients observe changes in the narratives through the narrative structure and content, in order internalise interpretations made about the narrative. This study adds to the emerging evidence that recorded recovery narratives have a strong potential to influence outcomes in individuals who have mental health concerns, and provides an evidence-based approach to informing evaluative processes in intervention trials.

## Supplementary information

**Additional file 1.** Characteristics of included recorded recovery narratives.

**Additional file 2.** Preliminary Coding Framework.

## Data Availability

The datasets generated and analysed during the current study are not publicly available as participants did not consent for their transcripts to be publically released. Extracts of participant responses have been made available within the manuscript.

## Abbreviations

GCSE: General Certificate of Secondary Education
GTAC: Gene Therapy Advisory Committee
HHI: Herth Hope Index
HTML: Hypertext Markup Language
ISRCTN: International Standard Randomised Controlled Trials Number
NECT: Narrative Enhancement Cognitive Therapy
NEON: Narrative Experiences Online
NVQ: National Vocational Qualification
REC: Research Ethics Committee
SD: Standard Deviation
