# Supplementary material for: The mechanisms and processes of connection: developing a causal chain model capturing impacts of receiving recorded mental health recovery narratives
Source: BMC Psychiatry. 2019 Dec 21;19:413. doi: 10.1186/s12888-019-2405-z (PMC6925452; doi:10.1186/s12888-019-2405-z)
Supplement: Supplementary file 1 — Additional file 1. Characteristics of included recorded recovery narratives. [file 12888_2019_2405_MOESM1_ESM.docx]

**Additional File 1.** Characteristics of included recorded recovery narratives

|  | **Narrator’s Age** | **Narrator’s Gender** | **Narrator Diagnosis** | **Modality** |
| --- | --- | --- | --- | --- |
| Story ID 1 | Not identifiable | Female | Mood-related | Text |
| Story ID 2 | Not identifiable | Female | Not identifiable | Text |
| Story ID 3 | 41-65 years | Male | Rejects diagnosis | Text |
| Story ID 4 | Not identifiable | Male | Rejects diagnosis | Text |
| Story ID 5 | Not identifiable | Female | Multiple: Mood-related and Substance-use | Text |
| Story ID 6 | Not identifiable | Female | Multiple: Mood-related and Substance-use | Text |
| Story ID 7 | Not identifiable | Female | Rejects diagnosis | Text |
| Story ID 8 | 41-65 years | Male | Multiple: Mood-related and Personality-related | Text |
| Story ID 9 | 41-65 years | Male | Schizophrenia and other psychosis | Text |
| Story ID 10 | 26-40 years | Female | Not identifiable | Text |
| Story ID 11 | 26-40 years | Female | Multiple: Mood-related and Schizophrenia and other diagnosis | Text |
| Story ID 12 | 41-65 years | Male | Multiple: Schizophrenia and other psychosis and Stress-related | Text |
| Story ID 13 | Not identifiable | Female | Mood-related | Text |
| Story ID 14 | Not identifiable | Not identifiable | Not identifiable | Text |
| Story ID 15 | 26-40 years | Female | Rejects diagnosis | Text |
| Story ID 16 | 0-25 years | Female | Multiple: Schizophrenia or other psychosis and stress related and rejects diagnosis | Video |
| Story ID 17 | 41-65 years | Male | Mood-related | Video |
| Story ID 18 | 41-65 years | Female | Not identifiable | Video |
| Story ID 19 | 26-40 years | Male | Schizophrenia and other psychosis | Video |
| Story ID 20 | 0-25 years | Male | Multiple: Neurodevelopmental and Mood-related | Video |
| Story ID 21 | 41-65 years | Male | Schizophrenia and other psychosis | Video |
| Story ID 22 | 0-25 years | Female | Multiple: Mood-related and Substance use | Video |
| Story ID 23 | 0-25 years | Female | Mood-related | Video |
| Story ID 24 | 26-40 years | Female | Mood-related | Video |
| Story ID 25 | 0-25 years | Female | Mood-related | Video |
| Story ID 26 | Not identifiable | Female | Mood-related | Audio |
| Story ID 27 | Not identifiable | Female | Not identifiable | Audio |
| Story ID 28 | Not identifiable | Male | Mood-related | Audio |
| Story ID 29 | Not identifiable | Male | Schizophrenia and other psychosis | Audio |
| Story ID 30 | 41-65 years | Male | Mood-related | Audio |
