# Supplementary material for: The mechanisms and processes of connection: developing a causal chain model capturing impacts of receiving recorded mental health recovery narratives
Source: BMC Psychiatry. 2019 Dec 21;19:413. doi: 10.1186/s12888-019-2405-z (PMC6925452; doi:10.1186/s12888-019-2405-z)
Supplement: Supplementary file 2 — Additional file 2. Preliminary Coding Framework. [file 12888_2019_2405_MOESM2_ESM.docx]

**Additional File 2. Preliminary Coding Framework**

**Mechanisms of Connection**

| **Sub-ordinate codes** | **Definition** | **NEON #5 (Systematic Review)** | **NEON #3 (Qualitative Study)** |
| --- | --- | --- | --- |
| **Mediators of Connection** | | | |
| Comparison of position in recovery | Comparison made on recovery position by the recipient, where the recipient feels they are doing better or worse than the narrator. | - Validation of self- through comparison of self to narrator and finds that they are doing well - Life circumstances and events in the narrative matched those of the recipient - The recipient is not at the right stage of recovery for a particular narrative to be beneficial | - Disconnection from others who have experienced recovery |
| Comparison of shared personal characteristics | Comparisons made by the recipient based on narrator characteristics. For example; gender, ethnicity, profession, diagnosis, economic situation, social status, severity of distress, frame of reference |  | - Shared characteristics – gender, ethnicity, profession, diagnosis, economic situation, social status, severity of distress, frame of reference, degree of recovery - Mismatch as a disruption to connection. Narrators who are very different to the recipient could lead to a negative impact. |
| Empathy | Feeling of empathy that leads to emotional connection or emotional responses from recipients | - The recipient feels empathy for the narrator and this initiates strongly felt emotional responses (feel movinged, sad, distressed, heartbroken, inspired, admiring) - The recipient has an empathic response to video-based recovery stories, making consumption tiring | - Emotional connection contributing to appreciation |
| Recognising shared experiences | The recipient identifies that the narrator has experienced similar experiences as themselves. | - The recipient feels reassured that others have experienced similar distress - Validation of self – through hearing views about illness and recognising these in themselves - The recipient is not able to relate to the narrator’s journey of recovery, leading to feelings of alienation and despair - The narrator presents specific details of their own behaviours or life events | - Feeling less alone - Feeling more like a community member - Feeling more connected to specific individuals - Validation - Disconnection from narrators experiencing less distress |
| Noticing narrator achievements | The recipient noticing the successes, strengths or survival of the narrator. |  | - Generates hope, empowerment, inadequacy and disconnection |
| Noticing narrator difficulties | The recipient notices the difficulties associated with the narrator’s recovery story. |  | - Generates appreciation, disconnection, pessimism and burden |
| Learning how recovery happens | The recipient develops new or further understanding about mental health and/or recovery from the narrator or narrative. | - Understanding severity, intensity and complexity of MH condition - How personal recovery might happen - Identifies personal behaviour they want to change - Learns from narrator who has experienced more profound difficulties | - Generates hope, empowerment and reference shift - Recovery as possible - Alternative conceptualisations of recovery - Strategies and barriers |
| Experiencing emotional release | The recipient experiences emotional responses as a result of engaging with the narrative. | - Empathy - Increased gratitude - Discomfort – difficult memories - Hope that recovery is possible - Anger – narrator experiences as insignificant - Pressure from narrator’s experiences | - Generates hope, reference shift and connectedness - ‘Bringing up stuff’ with narratives depicting distress |
| Avoidance of narratives | The recipient discontinues engagement with a recovery narrative | - The recipient avoids some recovery narratives due to the content being excessively challenging - The recipient feels significantly worse off than the narrator, and holds back from connection |  |
| **Moderators of Connection** | | | |
| Narrative authenticity | When the narrative is told in a manner which does not appear to be edited, that is it is a factual account from the narrator. | - The narrator appears to find it hard to express emotions or be authentic | - When narrative is told without apparent editing |
| Recipient in crisis | Recipient experiences crisis at the time of engaging with the narrative. |  | - Experiencing crisis can reduce the helpfulness of the narrative |
| Personal Beliefs | The world views or perspectives of recipients which may be in line with or contradict the views of the narrator. | - The recipient has strongly held personal beliefs that contradict with those of the narrator |  |
| No response | The recipient has no response after engaging with a narrative |  |  |

**Narrative influences of hope**

| **Sub-ordinate codes** | **Definition** | **NEON #5 (Systematic Review)** | **NEON #3 (Qualitative Study)** |
| --- | --- | --- | --- |
| Possibility of achievements | Hopefulness that recovery and achievements are possible. Achievements are defined as strength, success, survival. | - Recipient feels increased hope that recovery is possible | - Hopefulness about what achievements are possible |
| Optimism about human nature | Hopefulness through witnessing the positive personal characteristics of others |  | - Optimism about human nature ‘I think it is to find out they are such lovely people inside, like keep inside they are just normal people first of all… such a nice soul, they are wonderful, lovely people… very very inspiring’ [B020] |
| Pessimism about the possibility of recovery | Pessimism that recovery is possible |  | - Pessimism about possibility of recovery |
| Pessimism about problems of society | Pessimism surrounding the contribution of society to mental health concerns |  | - Pessimism about problems of society |
| Pessimism about the value of sharing narratives | Pessimism about the value of sharing recovery stories |  | - Pessimism about value of sharing narratives |
